# Glycoengineering CAR-T cells to overcome galectin-3-mediated immunosuppression

**DOI:** 10.3389/fimmu.2026.1766555

**Published:** 2026-02-18

**Authors:** Lee Seng Lau, Maria Suarez, Brandon Fernandez, Joseph Souchak, Aristotelis Antonopoulos, Nan Hu, Maria M. Abreu, Guenther Koehne, Anne Dell, Stuart M. Haslam, Avery D. Posey, Charles J. Dimitroff

**Affiliations:** 1 Department of Cellular and Molecular Medicine, Herbert Wertheim College of Medicine, Florida International University, Miami, FL, United States; 2 Department of Life Sciences, Imperial College London, London, United Kingdom; 3 Department of Wine, Vine & Beverage Sciences, School of Food Sciences, University of West Attica, Athens, Greece; 4 Department of Biostatistics, Robert Stempel College of Public Health and Social Work, Florida International University, Miami, FL, United States; 5 Miami Cancer Institute, Baptist Health-South Florida, Miami, FL, United States; 6 Department of Systems Pharmacology and Translational Therapeutics, Perelman School of Medicine, University of Pennsylvania, Philadelphia, PA, United States; 7 Corporal Michael J. Crescenz VA Medical Center, Philadelphia, PA, United States

**Keywords:** cancer glycobiology, cancer immunotherapy, CAR-T cells, galectins, sialylation modification

## Abstract

Chimeric antigen receptor (CAR)-T cell therapy has transformed treatment for relapsed or refractory B-cell malignancies; however, limited *in vivo* persistence and treatment-limiting toxicities continue to constrain durable efficacy. Because T cell glycan signatures and related galectin-binding properties impact their effector function, we postulated that CAR-T cells similarly displayed signature glycan features that govern their vulnerability to immunosuppressive galectins. In this report, public data mining, galectin-binding and glycosyltransferase expression assessments and glycomics showed that galectin (Gal)-3 was elevated in lymphoma-associated microenvironments and that anti-CD19 CAR-T cells displayed abundant Gal-3-binding glycans, reduced expression of the Gal-3–inhibitory enzyme α2,6-sialyltransferase 1 (*ST6GAL1*), and heightened susceptibility to Gal-3–mediated immunoregulation. To further explore this association, we enforced *ST6GAL1* expression in anti-CD19 CAR-T cells and found that Gal-3-binding was obstructed and Gal-3-mediated cell death and IL-5-induction were reversed. Enforcing *ST6GAL1* in CAR-T cells did not weaken tumoricidal activity and significantly improved anti-tumor responses and *in vivo* persistence. Collectively, this study identifies Gal-3 as a key extrinsic suppressor of CAR-T cell function and establish targeted cell surface α2,6 sialylation as a strategy to enhance CAR-T cell resistance to galectin-rich immunosuppressive microenvironments.

## 1 Introduction

Hematological malignancies remain a major cause of cancer-related morbidity and mortality, and despite advances in chemoimmunotherapy, durable responses are often limited in aggressive disease settings, including those treated with R-CHOP, polatuzumab-R-CHP, and dose-adjusted EPOCH-R (1, 2). Diffuse large B-cell lymphoma (DLBCL), the most common aggressive B-cell lymphoma, exemplifies these challenges, with a substantial proportion of patients developing relapsed or refractory disease (1, 3).

Therapeutic failure in refractory/relapse (r/r) DLBCL reflects both acquired drug resistance and immunosuppressive signals within the tumor microenvironment (TME) (4, 5). These challenges have driven the development of adoptive cellular therapies, most notably anti-CD19 chimeric antigen receptor (CAR) T-cell therapy, which is now established as a second-line standard of care for eligible DLBCL patients based on randomized clinical trials such as ZUMA-7 (axi-cel, NCT03391466) and TRANSFORM (liso-cel, NCT03575351) (6–8). Despite achieving deep remissions, CAR-T cell efficacy is frequently limited by insufficient *in vivo* persistence and exposure to immunosuppressive cues within the TME.

CAR-T cells are genetically engineered T cells that express a chimeric cell surface receptor designed to bind a target tumor antigen and, when engaged, transmit intracellular anti-tumor effector functions (9). Unlike T cells, CAR-T cells can recognize antigens independently of major histocompatibility complex (MHC) molecules, allowing for broader target recognition (10, 11). However, once infused, CAR-T cells encounter host- and tumor-derived immunoregulatory factors that impair survival and durability. Among these, galectins, a family of 15 β-galactoside–binding lectins, have emerged as key mediators of immune evasion in cancer (12, 13). Galectin-1, -3, and -9 suppress T-cell immunity by activating co-inhibitory pathways, disrupting co-stimulatory signaling, skewing cytokine production, and promoting apoptosis or exhaustion (14, 15). Galectin (Gal)-3, in particular, is highly enriched in lymphoma-associated microenvironments and has been shown to impair T-cell activation, differentiation, and effector function (14, 15). Importantly, galectin-mediated regulation is dictated not only by lectin abundance but also by the glycan architecture displayed on immune cells. Alpha 2,6 sialylation mediated by α2,6-sialyltransferase 1 (*ST6GAL1*) is a known inhibitor of Gal-3-binding, suggesting that changes in glycosyltransferase expression during CAR-T manufacturing may influence susceptibility to Gal-3-mediated immunosuppression.

Here, we investigated Gal-3 levels in DLBCL-associated microenvironments and examined the glycan landscape of end-of-manufacture anti-CD19 CAR-T cells, focusing on glycosyltransferase expression, Gal-3 ligand availability, and vulnerability to Gal-3-mediated immunoregulation. We further evaluated whether glycoengineering CAR-T cells to enforce ST6GAL1 expression could mitigate Gal-3-induced dysfunction and enhance CAR-T persistence and antitumor efficacy in a Gal-3-rich tumor microenvironments. This approach may provide a foundation for attempts to improve CAR-T cell durability and informing the development of optimized cellular immunotherapies.

## 2 Materials and methods

Complete details of the methods and reagents are available in **Supplementary Materials** and **Supplementary Table 1**.

### 2.1 ELISA of Gal-1, -3 and -9 in DLBCL patient sera

Sera were collected from 31 DLBCL patients and 31 healthy control donors from the Biospecimen Repository Facility at Baptist Health-South Florida/Miami Cancer Institute (Miami, FL) and stored at −80 °C until further analysis to assess Gal-1, -3, and -9 levels using a commercial ELISA kit (DGAL10, DGAL30, DGAL90; R&D Systems, Minneapolis, MN, USA). Absorbance was measured at 450nm, and a standard curve was generated using a Cytation 5 reader with Gen5 software (BioTek Instruments, Winooski, VT, USA). All samples were analyzed in triplicate. Gal-1, -3, and -9 levels in DLBCL patients were compared to those of healthy control donors, with results expressed in ng/ml. Complete details of reagents details are available in **Supplementary Table 1**. De-identified blood-derived serum samples were obtained from an institutional biospecimen repository. This study was reviewed by the Florida International University Office of Research Integrity and determined to be Not Human Subjects Research (NHSR) under IRB protocol IRB-22-0080. Blood sample collection and secondary use were conducted in accordance with Institutional Review Board approvals at FIU.

### 2.2 Gene expression data collection and processing

The GEO browser (https://www.ncbi.nlm.nih.gov/geo/browse/) was searched for microarray datasets GSE2350​ (comparing DLBCL (n = 11) to normal memory B cells (n = 5), naïve B cells (n = 5) and germinal center B cells (n = 17)) and GSE173263 (comparing early failure patients (n = 10) to responding patients (n = 29)) was analyzed using the interactive web tool GEO2R (https://www.ncbi.nlm.nih.gov/geo/geo2r/), which employs ‘limma’ package of R programming language for gene expression analysis (16). An adjusted *p* value < 0.05 was considered significant. ImmuCellAI (https://pmc.ncbi.nlm.nih.gov/articles/PMC7141005/) and the GSCA (Gene Set Cancer Analysis) (https://guolab.wchscu.cn/GSCA/#/) integrated databases were utilized to estimate the abundance of tumor-infiltrating immune cells in DLBCL samples (17). Statistical significance was determined using Spearman correlation with false discovery rate (FDR) correction (FDR ≤ 0.05).

### 2.3 Cell lines and cell culture

Human B-lymphoma Raji cell line expressing luciferase (Raji-Luc^+^) purchased from the ATCC (Manassas, VA). Human B-lymphoblast-like cell line (SUDHL-6-Luc^+^) was purchased from Creative Biolabs (Shirley, NY) were cultured in RPMI-1640/10% FBS. Cell cultures were regularly validated to be *Mycoplasma* free. Reagent details are available in **Supplementary Table 1**.

### 2.4 Plasmid amplification and lentiviral production

Anti-CD19 CAR (CD19 scFv/4-1BB/CD3ζ) and *ST6GAL1*/anti-CD19 (*ST6GAL1*-P2A-CD19 scFv/4-1BB/CD3ζ) (transfer plasmids) were kindly provided by Dr. Avery D. Posey Jr. The plasmids were amplified via heat shock transformation using Stbl3 cells (Invitrogen) and subsequently isolated with a Maxi Prep kit (QIAGEN) according to the manufacturer’s instructions. Lentiviral vectors were produced by transfecting HEK 293T cells (ATCC), which were grown to 70% confluency at 37 °C in a 5% CO_2_ incubator, with the transfer plasmids along with the LV-MAX Lentiviral mix (Gibco™, Waltham, MA, USA) and the lentiviral packaging plasmids—pRSV.REV (Rev expression vector), pMDLg/p.RRE (Gag/Pol expression plasmid, Addgene #12251), and pVSV-G (VSV glycoprotein expression vector)—were combined with Opti-MEM (Thermo Fisher Scientific) at a 15:18:18:7 mass-unit ratio. This DNA mixture was then added, at a 1:1 volume ratio, to a pre-warmed mixture of Opti-MEM and Lipofectamine 2000 (Thermo Fisher Scientific) and incubated at room temperature for 10 minutes before being added to the HEK 293T cells in fresh culture medium. Viral supernatants were collected at 24 and 48h post-transfection, passed through 0.45 μm filters, and concentrated using an Amicon^®^ Ultra Centrifugal Filter (Millipore). The concentrated lentivirus was stored at −80 °C, and the viral titer was determined using the Lenti-X™ Provirus Quantitation Kit (Takara Bio). Reagent details are available in **Supplementary Table 1**.

### 2.5 Isolation, transduction, and expansion of primary human T cells

Peripheral blood mononuclear cells (PBMCs) were isolated from normal healthy donor leukopacks (OneBlood, Inc., Miami, FL) using Ficoll-Paque (GE Healthcare) gradient centrifugation. PBMC aliquots were thawed, washed and subjected to immunomagnetic cell separation using the Pan T cell Isolation Kit and Naïve T cell Isolation Kit (Miltenyi Biotec). Naïve T cells were stimulated with ImmunoCult™ Human CD3/CD28 T Cell Activator (Stem Cell Technologies; 25 μl/ml) and transduced one day after activation with lentiviral vectors at a multiplicity of infection (MOI) of 3. To support T cell expansion, human IL-2 (R&D Systems) was added every other day for 10 days at a final concentration of 100 IU/ml. Day-10 expanded T cells refer to CD3/CD28-activated, IL-2–expanded T cells cultured for 10 days in parallel with CAR-T manufacturing but not subjected to lentiviral transduction. Reagent details are available in **Supplementary Table 1**.

### 2.6 RT-qPCR analysis

RT-qPCR analysis was conducted on RNA extracted from naïve T cells (control) and T cells activated for 2 days (Day 2), 5-days (Day 5) or for 10-days (Day 10), and CAR-T cell formulations. cDNA was synthesized, and real-time quantitative RT-PCR was performed to assess genes related to galectin ligand-building/suppressing ligand synthesis, *ST6GAL1, B4GALT1, B3GNT2, GCNT1, GCNT2, MGAT5* and *ST3GAL1*. Data was normalized to housekeeping gene 18S, and relative transcript levels were analyzed using the 2^(−ΔΔ^*Ct*^)^ method from 3 independent experiments (18). Reagent details are available in **Supplementary Table 1**.

### 2.7 Flow cytometry

Flow cytometry assays incorporated cell staining reagents: live/dead cell marker Zombie, 7-AAD (Biolegend). For assessment of endogenous Gal-3 expression on target cells, cells were stained with an anti–Gal-3 antibody (BioLegend) together with an isotype-matched control antibody to define background signal. For recombinant galectin binding, recombinant human (rh) Gal-1, -3 and -9 (All from R&D Systems), anti-Gal-1, -Gal-3, and -Gal-9 Abs (Biolegend) as well as Abs to differentiation markers, CD3, CD4 and CD8. Cells were stained with rhGal-1, -3 and -9 at 3µg/ml, 5µg/ml and 1µg/ml, respectively, as we report (19–22), with or without 50mM lactose to confirm galactose-binding dependency, and then stained with respective anti-Gal Ab or isotype-matched control Ab. Plant lectin staining was performed using *Sambucus nigra* agglutinin (SNA; Vector Laboratories) to detect α2,6-linked sialylation, *Maackia amurensis* lectin II (MAL II; Vector Laboratories) to detect α2,3-linked sialylation, and *Solanum tuberosum* lectin (STL; Vector Laboratories) to detect poly-LacNAc residues. Cells were washed with PBS containing 1% BSA and stained on ice with the respective biotinylated lectins, along with a live/dead marker (Zombie; Biolegend) followed by FITC (Biolegend). CAR detection was performed using biotin-conjugated goat anti-mouse F(ab′)_2_ antibody (Jackson ImmunoResearch) followed by PE-streptavidin (Biolegend). Singlets were gated using FSC-H vs. FSC-A and SSC-H vs. SSC-A, and lymphocytes were identified based on forward/side scatter. Stained cells were immediately acquired using BD FACS Diva 6.1 software and analyzed in FlowJo (BD Biosciences). Data were reported as mean fluorescence intensity (MFI ± SEM). Background fluorescence were controlled using appropriate negative controls depending on the assay. For galectin binding experiments, samples stained in the presence of 50 mM lactose were used as competitive binding controls, and mean fluorescence intensities (MFI) from lactose-treated samples were subtracted from experimental values. For antibody-based staining, isotype-matched control antibodies and/or unstained controls were used to define background signal, and corresponding MFIs were subtracted prior to data analysis. Reagent details are provided in **Supplementary Table 1**. All experiments were performed 3-times to obtain statistical significance.

### 2.8 Cell proliferation assay

Cell proliferation was evaluated using the Cell Counting Kit-8 (CCK-8; Dojindo, Japan) following the manufacturer’s protocol. Cells were seeded in 96-well plates at a density of 2,000 cells per well. Proliferation was assessed at 0, 24, 48, and 72 hours by adding 10 µL of CCK-8 reagent to each well and incubating at 37 °C for 3 hours. Absorbance was measured at 450 nm using a Cytation 5 microplate reader (BioTek, Winooski, VT, USA). All experiments were performed in 3-times to ensure statistical significance.

### 2.9 MALDI-TOF MS N-glycomic analysis of human T cells

CAR-T cells and control T cell cohorts were treated as described previously (22). Cell pellets were sonicated in ice-cold lysis buffer (25 mM Tris, 150 mM NaCl, 5 mM EDTA, and 1% CHAPS, pH 7.4), dialyzed in Slide-A-Lyze™ dialysis cassettes (3.5 kDa cutoff; Thermo), reduced by DTT in 4 M guanidine-HCl (Thermo), carboxymethylated by IAA, and then digested by trypsin (Sigma, 24 h, 37 °C). The digested samples were purified using Oasis plus short HLB Sep-Pak (Waters) extraction cartridges. N-glycans were released by PNGase F (recombinant from *Escherichia coli*, E.C. 3.5.1.52; Roche) digestion (24 h, 37 °C) and purified by classic short C18-Sep-Pak (Waters). Released N-glycans were permethylated with methyl iodide and then purified by classic short C18-Sep-Pak (Waters) prior to MS analysis. All permethylated glycans were dissolved in 10 μl methanol. One 1 μl of sample was mixed with 1 μl of matrix (10 mg/ml 3,4-diaminobenzophenone (Acros Organics) in 75% (v/v) aqueous acetonitrile) and spotted onto a MALDI plate. MS data were acquired using a 4800 MALDI-TOF (Applied Biosystems, Darmstadt, Germany) mass spectrometer. The 4700 calibration standard kit, Calmix (Applied Biosystems) was used as the external calibrant for MS.

Data were analyzed using Data Explorer™ (version 4.9, Applied Biosystems) and GlycoWorkBench (23). The glycomic data were annotated based on monosaccharide composition derived from the molecular ion m/z value, knowledge of glycan biosynthetic pathways, the isotopic peak cluster patterns, and MS derived fragmentation (24).

### 2.10 Cell death assays

To investigate the effect of Gal-3 on apoptotic induction, T cells (1×10^5^/mL) were cultured in 96-well plates with rhGal-3 (R&D systems) (4µg/mL ± 50mM lactose) for 16h (25). Cells were stained with FITC-annexin-V and PI, then analyzed by flow cytometry to determine viable (annexin-V^-^ PI^-^), early apoptotic (annexin-V^+^ PI^-^), and late apoptotic/necrotic (annexin-V^+^ PI^+^) populations. All experiments were performed 3-times to obtain statistical significance. Reagent details are available in **Supplementary Table 1**.

### 2.11 Cytokine profiling arrays

Cells were treated with rhGal-3 (4µg/ml) with/without 50mM lactose for 16h. Supernatants were collected and pro-/anti-inflammatory cytokines and chemokines, C5a, CD40L, G-CSF, GM-CSF, CXCL1, IL-8, IL-16, IL-18, CCL5, IL-1ra, SerpinE1, IL-1α, IL-1β, IL-2, IL-4, IL-5, IL-6, IL-10, IL-12p70, IL-13, IL-17, IL-17E, IL-23, IL-27, IL-32α, MIF, ICAM-1, IFN-γ, CXCL10, CXCL11, CCL2, CCL3, CCL4, CXCL12, TNF-α and TREM-1, were measured using a Proteome Profiler Human Cytokine Array Kit (R&D systems), according to the manufacturer’s instructions. Streptavidin-HRP detection agents was subsequently used to develop the blot with the intensity of the spot in direct proportion to the amount of cytokine bound. Membranes were visualized with the iBright™ FL1500 Imaging System (Invitrogen) and analyzed using iBright™ Analysis Software (Invitrogen). All experiments were performed 3-times to obtain statistical significance. Reagent details are available in **Supplementary Table 1**.

### 2.12 Intracellular IL-5 analysis

To investigate the effect of Gal-3 on cytokine induction, cells were incubated with 4µg/ml rhGal-3 (R&D systems) with 200x10^3^/ml CAR-T cells, ST6^OE^ CAR-T cells, and Day 10-expanded T cells (control) in complete medium in 96-well plates for 16h in 200 µL at 37 °C. For 4-6h, cells were activated with a pre-mixed cocktail containing PMA, ionomycin, and Brefeldin A (BD Biosciences) to block cytokine secretion. Cells were then washed twice with PBS buffer and stained with Zombie Aqua™ Fixable Viability (Biolegend) for 20 min at room temperature. Cells were then washed in cell staining buffer (BD Biosciences) following immunostaining with a surface stain cocktail containing the following antibodies: CD8a PerCP/Cyanine5.5 (Biolegend), CD4 BV605 (Biolegend), and CD3ϵ APC/Fire 750 (Biolegend) in Stain Buffer (BD Biosciences). After 30 min of incubation, cells were washed twice and incubated with 500 µL of BD Fixation and Permeabilization Solution (BD Biosciences) at 4 °C for 20 min, washed twice with BD Perm/Wash Buffer (BD Biosciences), and stained with a cocktail of fluorescently labeled anti-IL-5 APC (Biolegend) in Stain Buffer (BD Biosciences). After 30 min, the cells were washed once with 1× Perm/Wash Buffer (BD Biosciences) and resuspended in 200 µL of Stain Buffer (BD) prior to flow cytometric analysis. All experiments were performed 3-times. Reagent details are available in **Supplementary Table 1**.

### 2.13 Tumoricidal assay

To evaluate tumoricidal activity and the effect of IL-5 in tumoricidal activity, T cells were all incubated +/- 4µg/ml rhGal-3, +/- 1 or 10 ng of rhIL-5 in co-cultures of human CD19^+^ Raji-luc^+^ or SUDHL-6-luc^+^ target cells in 96-well plates (VWR) RPMI-1640/10% FBS. Effector: Target (T cell:tumor cell-luc^+^) at 20:1 for a 16h incubation in a total volume of 100 µL RPMI-1640/10% HI-FBS. Post-incubation, T cell and tumor cell-luc^+^ co-cultures were lysed using Bright-GLO reagent (Promega) and/or Nano-Glo Live Cell Assay System (Promega), according to the manufacturer’s instruction. Luminescence were analyzed using AMI HT imaging system. Gal-3^high^ and Gal-3^low^ target cell designations were defined based on relative endogenous Gal-3 expression assessed by flow cytometry under standard culture conditions prior to tumoricidal assays. SUDHL-6-luc^+^ cells consistently exhibited higher Gal-3 expression compared with Raji-luc^+^ cells across independent experiments and were designated Gal-3^high^, whereas Raji-luc^+^ cells were designated Gal-3^low^. All experiments were performed 3-times to obtain statistical significance. Reagent details are available in **Supplementary Table 1**.

### 2.14 *In vivo* anti-tumor assays

To compare anti-tumor efficacy of ST6^OE^ CAR-T cells with control CAR-T cells, we inoculated equal numbers of 6–8-week-old male/female NOD-SCID IL-2Rγ-deficient (NSG) mice intravenously (i.v.) with 10^5^ Raji-luc^+^ cells (100µl media). After 7 days, media or T cell cohorts (5×10^6^ in 100µl media) were i.v. transferred. Tumor burdens were assayed weekly for 35 days by i.p. administration of 100μl of 15mg/ml D-luciferin solution (VivoGlo™Luciferin)(Promega) and bioluminescent imaging (BLI) with AMI HT imager. For BLI, mice were anesthetized with 2% isoflurane and maintained under anesthesia by continuous inhalation of isoflurane until imaging was complete. Ventral and lateral images of the mice were taken 10 min later using AMI HT imaging system and quantified using the Aura Imaging Software. Tumor growth and survival were analyzed using growth rates and Kaplan-Meier curves (26, 27). All experimental procedures were conducted as per Florida International University Institutional Animal Care and Use Committee (IACUC) guidelines and IACUC protocol # IACUC-25-049-AM02.

### 2.15 Statistical analysis

Prism 8.0 software (GraphPad) was used for statistical analysis Data distribution was assessed using the Shapiro–Wilk normality test. For comparisons between two groups with non-normally distributed data, the Mann–Whitney U test was used, and Student’s t-test was applied when normality assumptions were met. For comparisons involving more than two groups, the Kruskal–Wallis test followed by Dunn’s multiple-comparisons correction was applied. For experiments involving two independent variables and repeated measurements, data were analyzed using a two-way repeated-measures ANOVA with Sidak’s multiple-comparisons test. To account for potential age-related confounding, age-adjusted analyses were performed using multiple linear regression, with disease status(healthy vs DLBCL) and age included as independent variables. Regression analyses were conducted in Prism using one observation per individual. For survival analysis, Gehan-Breslow-Wilcoxon test was used. Throughout, data are presented as the means SEM, unless otherwise noted. A *p* value of *p* < 0.05 was considered statistically significant. For transcriptomic datasets and immune-infiltration analyses (ImmuCellAI, GSCA), multiple-testing correction was performed using Spearman’s rank correlation, with statistical significance determined following false discovery rate (FDR) correction using the Benjamini–Hochberg method (FDR ≤ 0.05).

## 3 Results

### 3.1 Elevated Gal-3 levels are associated with DLBCL patient serum, tumor-intrinsic expression, and macrophage-rich TME

**Figure 1:**
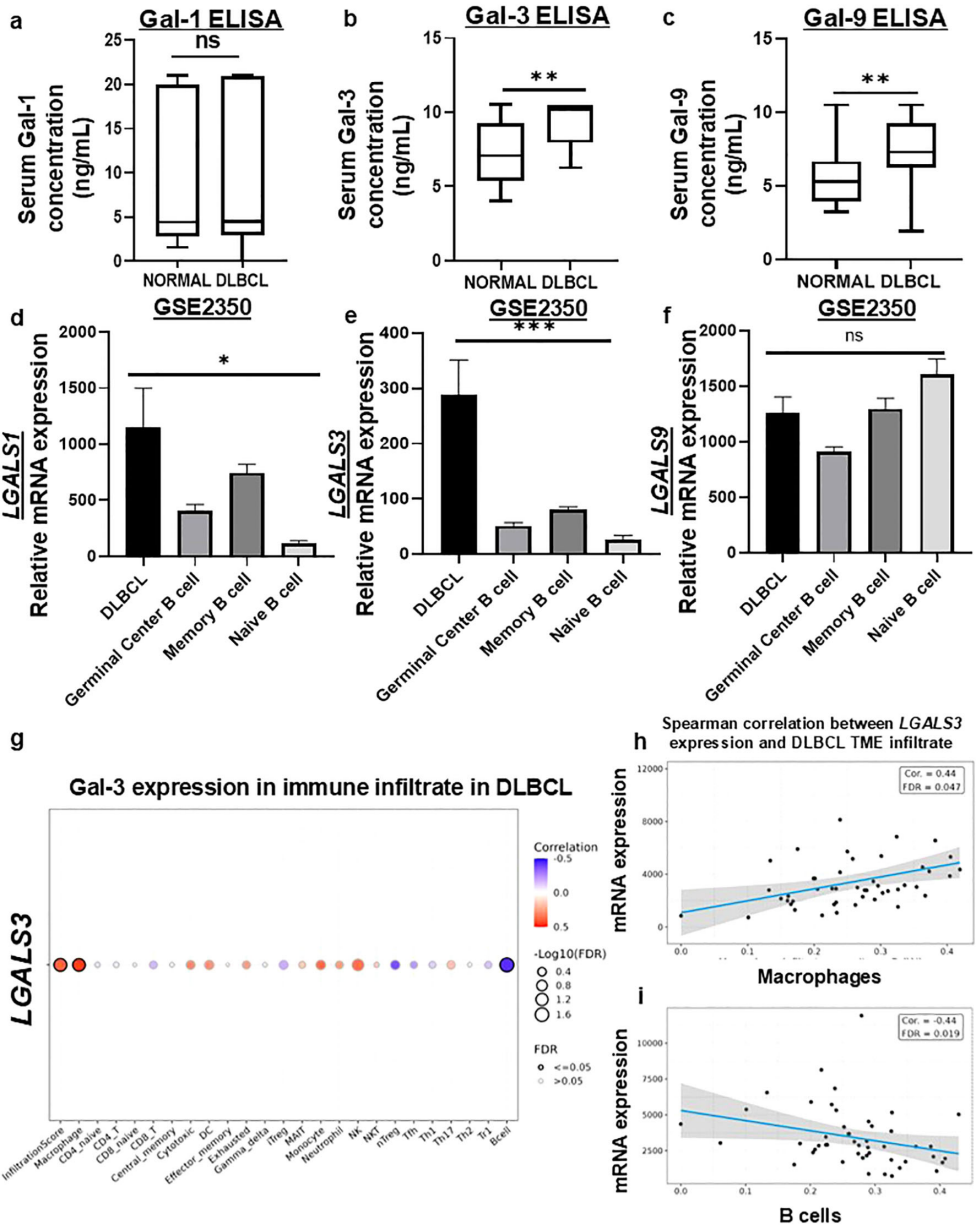
Gal-3 is elevated in DLBCL patients, DLBCL tumor cells, and DLBCL tumor-infiltrating macrophages. ELISA of serum Gal-1 **(a)**, -3 **(b)**, and -9 **(c)** levels depicted significantly higher circulating levels of Gal-3 and -9 in DLBCL patients, compared to healthy individuals after using multiple linear regression with disease status and age included as covariance (**p < 0.01). Transcriptional expression analysis of GEO GSE2350 datasets shows elevated expression of Gal-1 and Gal-3 in DLBCL cells compared to healthy B cell subsets (*p<0.05, ***p<0.001) **(d)**, *LGALS1* **(e)** *LGALS3* and **(f)** *LGALS9.* Analysis of immune cell infiltrates within DLBCL samples retrieved from TCGA datasets revealed **(g)** correlations and infiltration score of *LGALS3* expression between 24 immune infiltrates. A positive spearman correlation was observed between *LGALS3* expression and macrophage infiltration **(h)** and overall immune infiltration score, whereas a negative correlation was observed between LGALS3 and B cell infiltration **(i)**. Statistical significance was determined using Spearman correlation with false discovery rate (FDR) correction (FDR ≤ 0.05).

To assess whether immunosuppressive galectins are enriched in DLBCL-associated disease settings, we analyzed serum levels of Gal-1, -3, and -9 in 31 DLBCL patients and 31 age-matched healthy controls (**Supplementary Table 2**). Serum Gal-1 levels showed no significant difference between groups (**Figure 1a**). In contrast, DLBCL patients exhibited significantly elevated levels of Gal-3 (7.17 ± 0.50ng/mL vs. 9.07 ± 0.50ng/mL, *p* < 0.01) (**Figure 1b**) and Gal-9 (5.468 ± 0.53ng/mL vs. 7.183 ± 0.53ng/mL, *p* < 0.01) (**Figure 1c**) compared to healthy individuals after adjusting for age using multiple linear regression. Age was also found as an independent predictor of Gal-3 levels (*p* < 0.001). These findings indicate that circulating Gal-3 and Gal-9 are elevated in DLBCL-associated disease states.

To evaluate tumor-associated galectin expression, we then analyzed the microarray dataset GSE2350 generated on Affymetrix U95 (GL91) platform and compared *LGALS1*, *LGALS3*, and *LGALS9* transcription in DLBCL samples (n=11) with normal germinal center B cells (n=17), memory B cells (n=5), and naïve B cells (n=5) (**Figures 1d–f**). Within this context, Gal-1 (*p* < 0.05) and Gal-3 (*p* < 0.001) were significantly upregulated in DLBCL relative to normal mature B cell subsets (**Figures 1d, e**), indicating altered galectin expression within lymphoma tissues. Next, we interrogated the RNA-seq dataset GSE173263 to compare galectin expression between DLBCL patients with early treatment failure (primary refractoriness or relapse within 12 months; n = 10) and patients achieving remission (n = 29) (**Supplementary Figure 1a**) (28). Although Gal-1, Gal-3, and Gal-9 transcripts trended higher in early-failure patients, these differences did not reach statistical significance (**Supplementary Figure 1b-d**), suggesting that galectin expression alone may not stratify clinical outcomes.

To assess potential cellular sources of Gal- within the tumor microenvironment, we analyzed *LGALS3* mRNA expression across immune cell subsets using ImmucellAI and GSCA with TCGA DLBCL datasets (**Figure 1g**). *LGALS3* expression correlated positively with macrophage abundance and overall immune infiltration scores (FDR ≤ 0.05) (**Figure 1h**), while exhibiting a negative correlation with B-cell infiltration (FDR ≤ 0.05) (**Figure 1i**). These findings suggested that, in addition to tumor cell-intrinsic expression, Gal-3 may be contributed by macrophage-rich TME.

### 3.2 Anti-CD19 CAR-T cells displayed enhanced Gal-1- and Gal-3-binding and a glycosyltransferase expression program favoring galectin ligand synthesis

**Figure 2:**
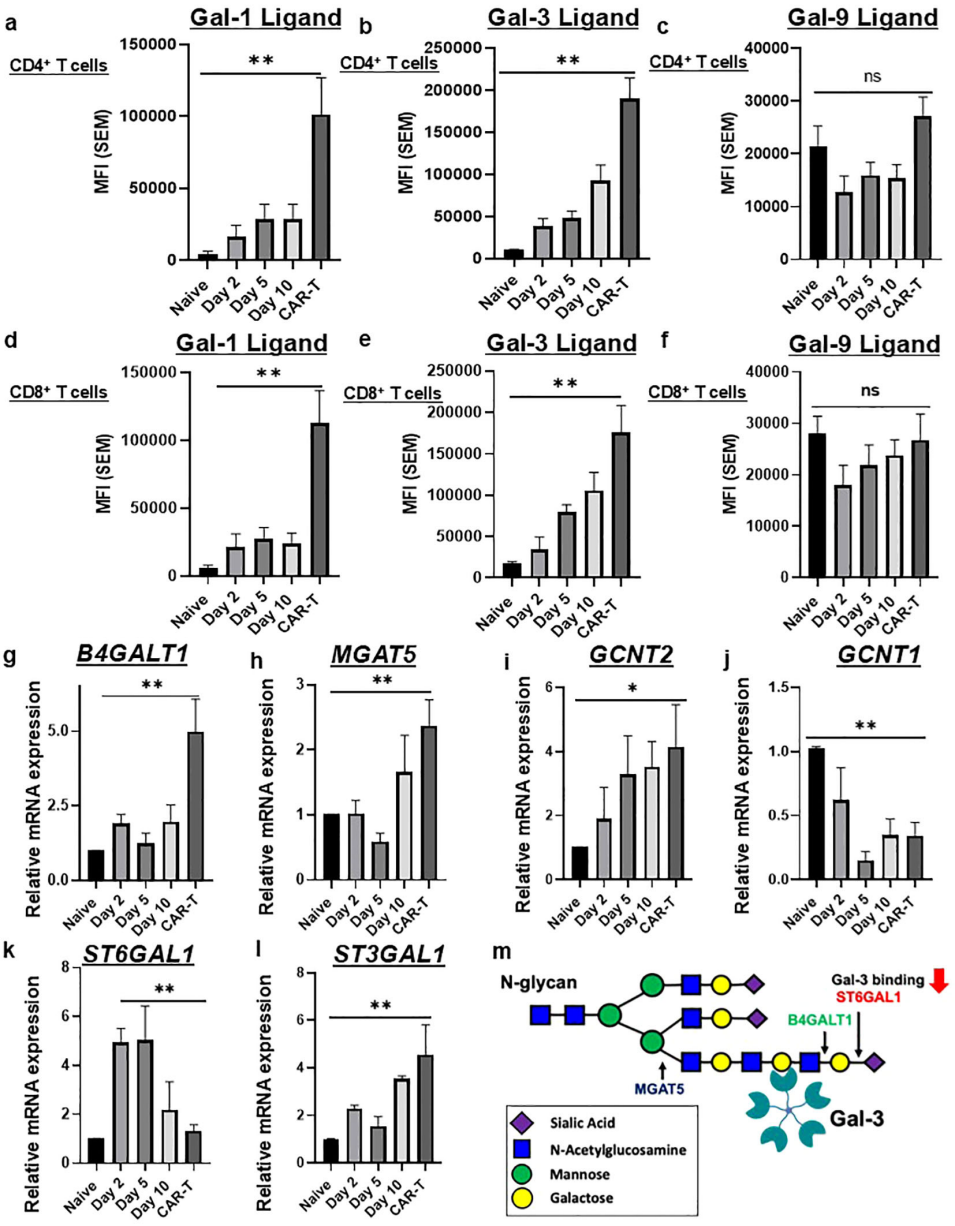
Anti-CD19 CAR-T cells avidly bound Gal-1 and -3 and, correspondingly, expressed high levels of Gal-1 and -3 glycan ligand-building enzymes, *B4GALT1* and *MGAT5*, and low levels of Gal-1 and -3 glycan ligand-inhibitory enzyme, *ST6GAL1*. Naïve T cells and corresponding CD4^+^ or CD8^+^ T cell isolates from PBMC were activated using CD3/CD28 microbeads. Cells were stained with Abs to T cell markers (CD3, CD4 and CD8) and Gal-1,Gal-3, and Gal-9 ligands were assessed by rhGal-1, rhGal-3, and rhGal-9 **(a–f)**, staining. Binding controls consisted of 50mM lactose in the buffers and the mean fluorescence intensities (MFI) of Gal stains were subtracted from lactose control. Data are graphically presented as MFI ± (SEM) on Day 0 (naïve), Day 2 (activated T cells), Day 5- and Day 10-expanded T cells (denote CD3/CD28-activated, IL-2–expanded, non-transduced T cells cultured in parallel with CAR-T cells) and CAR-T cells from at least 6 independent donors. Statistical analysis was performed using a Kruskal–Wallis test with Dunn’s multiple-comparisons correction (**p < 0.01). RT-qPCR analysis *of* **(g)** *B4GALT1*, **(h)** *MGAT5*, **(i)** *GCNT2*, **(j)** *GCNT1*, **(k)** *ST6GAL1, and* **(l**) *ST3GAL1* was performed on Day 0 (naïve T cells), Day 2 (activated T cells), Day 5- and Day 10-expanded T cells, and CAR-T cells. Graphed expression data from ≥5 donors are presented as Fold Change ± (SEM) of control Day 0 (naïve T cells). Statistical analysis was performed using a Kruskal–Wallis test with Dunn’s multiple-comparisons correction (**p < 0.01, *p < 0.05). Data were normalized to the housekeeping gene 18s. **(m)** Model illustrating how elevated *MGAT5*/*B4GALT1*-driven glycan branching and elongation, together with suppressed ST6GAL1 expression, promotes Gal-3 ligand formation. MFIs were background-corrected by subtraction of the appropriate negative control (lactose, isotype, or unstained).

To assay CAR-T cell binding to Gal-1, -3 and -9, flow cytometric analysis with recombinant human (rh) Gal-1, -3 and -9 and anti-Gal antibodies (Abs) was performed over a 10-day course of T cell activation/expansion and anti-CD19 CAR-T cell (anti-CD19 scFv/4-1BB/CD3ζ) manufacturing process from healthy donors. Negative controls included staining with isotype-matched Abs in place of anti-Gal Abs or staining with rhGal and anti-Gal Abs in the presence of lactose, a competitive inhibitor of galectin binding (**Supplementary Figure 1e-g**). CAR-T cells exhibited significantly increased Gal-1 and Gal-3 binding to both CD4^+^ and CD8^+^ subsets (**Figures 2a–e**). In contrast, Gal-9-binding did not differ significantly between CAR-T and naïve T cells (**Figures 2c, f**), indicating selective enhancement of Gal-1 and Gal-3 ligand availability following CAR-T cell manufacturing.

To investigate the glycome basis for enhanced galectin binding, we assessed expression of key glycosylation enzymes in CAR-T cells in comparison with T cell controls (**Figures 2g–l**). RT-qPCR analysis revealed significant elevations in β1,4 galactosyltransferase 1 (*B4GALT1*) (**Figure 2g**) (*p* < 0.01), ß1,6 N-acetylglucosaminyltransferase V (*MGAT5*) (**Figure 2h**) (*p* < 0.01), and core 2 β1,6 N-acetylglucosaminyltransferase 2 (*GCNT2)* (**Figure 2i**) (*p* < 0.05). These enzymes promote increased N-glycan branching and antennae extension with N-acetyllactosamine (LacNAc) units, which confer high affinity galectin binding. In contrast, expression of core 2 β1,6 N-acetylglucosaminyltransferase 1 (*GCNT1*) (**Figure 2j**) (*p* < 0.01) and β-galactoside α2,6 sialyltransferase 1 (*ST6GAL1*) (**Figure 2k**) (*p* < 0.01) were markedly reduced in CAR-T cells. *ST6GAL1* action caps terminal ß-galactose residues with α2,6-linked sialic acid to block Gal-1 and Gal-3 ligand recognition (29), while *GCNT1* generates core 2 O-glycans that can be elongated into galectin-binding LacNAc structures. Interestingly, expression of β-galactoside α2,3 sialyltransferase 1 (*ST3GAL1*) (**Figure 2l**) (*p* < 0.01) was increased in CAR-T cells; so, together with *GCNT1* loss, this expression pattern likely resulted in α2,3 sialylated core 1 O-glycans that are poor galectin ligands. Together, these results indicated that the Gal-1 and Gal-3 ligand-generating glycome of CAR-T cells was represented by a *GCNT1*^low^/*ST3GAL1*^high^ O-glycan program and an N-glycan program driven by upregulated expression of *MGAT5*/*B4GALT1*/*GCNT2* branching/elongation enzymes combined with downregulated expression of *ST6GAL*, consistent with increased Gal-1 and Gal-3 ligand availability (**Figure 2m**).

### 3.3 Anti-CD19 CAR-T cell N-glycome was represented by high N-glycan branching/elongation with concomitant reduction in α2,6 sialylation

**Figure 3:**
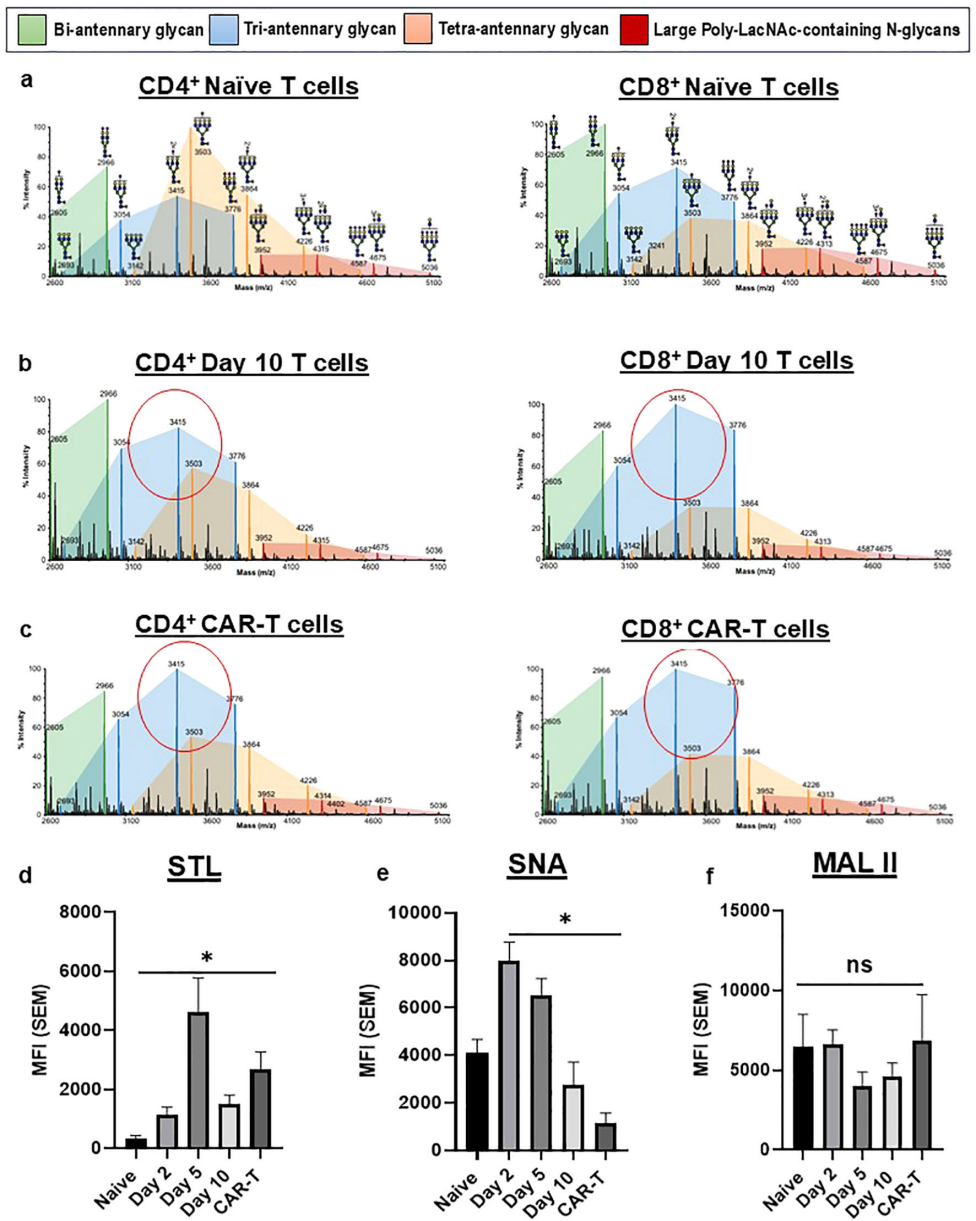
Anti-CD19 CAR-T cells displayed elevations in tri-antennary N-glycans and poly-N-acetyllactosamines (poly-LacNAc) and low levels of α2,6 sialylation. MALDI-TOF/TOF MS spectra of permethylated N-glycans of **(a)** CD4^+^ and CD8^+^ naïve T cells, **(b)** CD4^+^ and CD8^+^ Day10-expanded T cells and **(c)** CD4^+^ and CD8^+^ anti-CD19 CAR-T cells. T cell controls and CAR-T cells were stained with plant lectins STA **(d)**, which binds poly-LacNAc, SNA **(e)**, which binds α2,6-linked sialic acids, and MAL-II **(f)**, which binds α2,3-linked sialic acids. Data are graphically presented as MFI ± (SEM) on Day 0 (Naïve), Day 2 (activated T cells), Day 5- and Day 10-expanded T cells and CAR-T cells from at least 4 independent donors. Statistical significance was determined using a Kruskal–Wallis test with Dunn’s multiple-comparisons correction (*p < 0.05).

Profiling of cell N-glycans by Matrix-Assisted Laser Desorption/Ionization-Time of Flight mass spectrometry (MALDI-TOF MS) revealed an enrichment of highly branched N-glycan structures in anti-CD19 CAR-T cells. As shown in the mass spectra (**Figure 3a**), naïve T cells predominantly displayed smaller, bi-antennary complex N-glycans (green-shaded peaks); whereas Day 10 non-transduced expanded T cells (**Figure 3b**) and CAR-T cells (**Figure 3c**) displayed greater peaks corresponding to tri-antennary N-glycans (blue-shaded peaks). Tri-antennary N-glycans contain poly-N-acetyllactosamine (poly-LacNAc) chains that serve as glycan ligands for Gal-1 and Gal-3 (12), providing a structural basis for elevated Gal-1 and Gal-3 binding capacity.

Cell surface staining with plant lectins corroborated MS N-glycomics structural findings, showing that, compared with naïve T cells, anti-CD19 CAR T cells significantly bound Solanum tuberosum (potato) lectin (STL), which specifically recognizes poly-LacNAc motifs (p<0.05) (**Figure 3d**). Conversely, binding of Sambucus nigra agglutinin (SNA), which recognizes α2,6 sialylated LacNAc on N-glycans, was markedly reduced on CD19 CAR-T cells (p<0.05) (**Figure 3e**). This loss of SNA reactivity was consistent with the downregulation of ST6GAL1 and fewer Gal-1 and Gal-3 ligand inhibitory α2,6-sialylated LacNAc caps on CAR-T cell N-glycan antennae. Additionally, Maackia amurensis lectin II (MAL II), which binds α2,3 sialylated LacNAc, showed no significant difference between CAR-T cells and naïve T cells, suggesting that overall α2,3 sialylation levels were largely maintained on CAR-T cells (**Figure 3f**). These lectin-binding results together with MALDI-TOF MS data illustrated that, relative to naïve and non-transduced expanded T cell controls, CAR-T cells displayed a signature glycome enriched for poly-LacNAc structures on multi-antennary N-glycans with diminished α2,6 sialylation.

### 3.4 Gal-3 binding to anti-CD19 CAR-T cells promoted cell death, whereas enforced ST6GAL1 expression in anti-CD19 CAR-T cells diminished Gal-3-binding and related cell death

**Figure 4:**
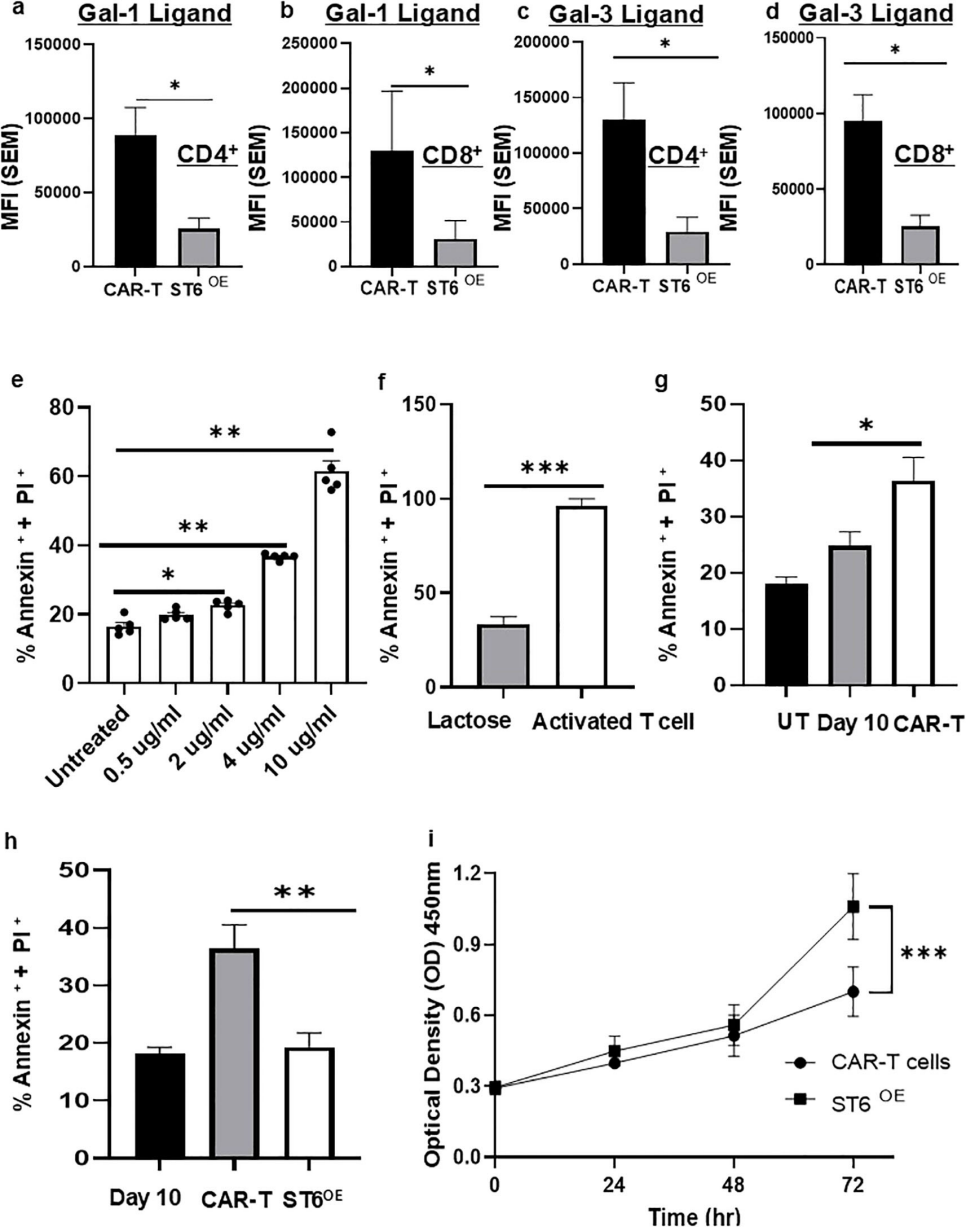
ST6^OE^ CAR-T cells exhibited suppressed Gal-1 and -3 ligand activity and resist Gal-3-mediated cell death. Anti-CD19 CAR-T cells and ST6^OE^ CAR-T were stained with rhGal-1 **(a)** CD4^+^ and **(b)** CD8^+^ and rhGal-3 **(c)** CD4^+^ and **(d)** CD8^+^ to assessed ligand binding via flow cytometry. Data represents n = 6 independent donors. Statistical significance was determined using a Mann–Whitney U test (*p < 0.05). **(e)** Gal-3 dose-response analysis showing a concentration-dependent increase in T-cell apoptosis following overnight incubation with increasing concentrations of rhGal-3 (0.5–10 µg/mL), quantified as Annexin V^+^/PI^+^ cells. **(f)** Activated T cells incubated overnight with rhGal-3 (4µg/mL) exhibited significantly increased Annexin V^+^/PI^+^ staining compared with lactose-treated controls. **(g)** Comparison of Gal-3–induced apoptosis between untreated (UT) Day 10–expanded T cells and Day 10 anti-CD19 CAR-T cells following overnight rhGal-3 exposure. **(h)** Comparison of Gal-3–induced apoptosis between Day 10 anti-CD19 CAR-T cells and ST6^OE^ CAR-T cells following overnight rhGal-3 exposure, demonstrating reduced Annexin V^+^/PI^+^ staining in ST6^OE^ CAR-T cells. For panels **(e–h)**, Annexin V^+^/PI^+^ values are presented as mean ± SEM from ≥4 independent donors, and statistical significance was determined using a Mann–Whitney U (*p < 0.05, **p < 0.01, ***p < 0.001). MFIs were background-corrected by subtraction of the appropriate negative control (lactose, isotype, or unstained). **(i)** Baseline proliferation of anti-CD19 CAR-T cells and ST6^OE^ CAR-T cells assessed by CCK-8 assay over 72 (h) Data are presented as optical density (OD 450 nm) ± SEM from n = 4 independent donors and analyzed using a two-way repeated-measures ANOVA with Sidak’s multiple-comparisons test (*p < 0.001).

To examine the role of ST6GAL1/α2,6 sialylation expression in modulating Gal-3 binding activity, we generated ST6GAL1/anti-CD19 CAR-T cells (ST6OE CAR-T cells) using a dual ST6GAL1-CD19 CAR (ST6GAL1-P2A-anti-CD19 scFv/4-1BB/CD3ζ) construct. Compared with control CAR-T cells, ST6OE CAR-T cells, which were routinely verified to display increased α2,6 sialylation using SNA-binding (**Supplementary Figure 2a, b**), exhibited significantly reduced rhGal-1 and rhGal-3 binding on both CD4^+^ and CD8^+^ subsets (**Figures 4a–d**) (p < 0.05), indicating that enforced ST6GAL1 expression can modify the glycan landscape of CAR-T cells to reduce Gal-1 and Gal-3 ligand activity. This reduction in Gal-1- and Gal-3-binding suggested that ST6OE CAR-T cells may be less prone to Gal-1- and Gal-3-mediated immunosuppression.

Given the enrichment of Gal-3 within DLBCL-associated tumor microenvironments, we next investigated whether elevated Gal-3 binding sensitizes anti-CD19 CAR-T cells to Gal-3–mediated cell death. Gal-3 dose-response analyses demonstrated that increasing concentrations of rhGal-3 induced a dose-dependent increase in T-cell apoptosis, as measured by Annexin V and PI (markers of early and late apoptosis, respectively) (**Figure 4e**). Consistent with glycan-dependent Gal-3 activity, activated T cells treated overnight with 4 µg/mL rhGal-3 exhibited significantly increased Annexin V^+^/PI^+^ staining compared with lactose-treated controls (**Figure 4f**) (*p* < 0.001), in line with prior reports demonstrating extracellular Gal-3–induced T-cell death via glycan binding (30). Compared with non-transduced Day-10–expanded T cells (CD3/CD28-activated, IL-2–expanded), CD19 CAR-T cells were more sensitive to rhGal-3-induced cell death (**Figure 4g**) (*p* < 0.05) . To evaluate the role of *ST6GAL1* in mitigating Gal-3-mediated apoptosis, we compared Gal-3-induced cell death of ST6^OE^ CAR-T cells vs. control CAR-T cells and found that ST6^OE^ CAR-T cells showed a significant decrease in Annexin V^+^/PI^+^ staining, demonstrating that *ST6GAL1* expression conferred protection against Gal-3-induced apoptosis (**Figure 4h**) (*p* < 0.01). Furthermore, baseline proliferation by CCK-8 assay showed ST6^OE^ CAR-T cells exhibited significant enhanced proliferative capacity after 72hr compared with conventional CAR-T cells, indicating improved intrinsic expansion potential (**Figure 4i**) (*p* < 0.01).

### 3.5 Gal-3 induced IL-5 secretion in anti-CD19 CAR-T cells, which was suppressed by enforced ST6GAL1 expression

**Figure 5:**
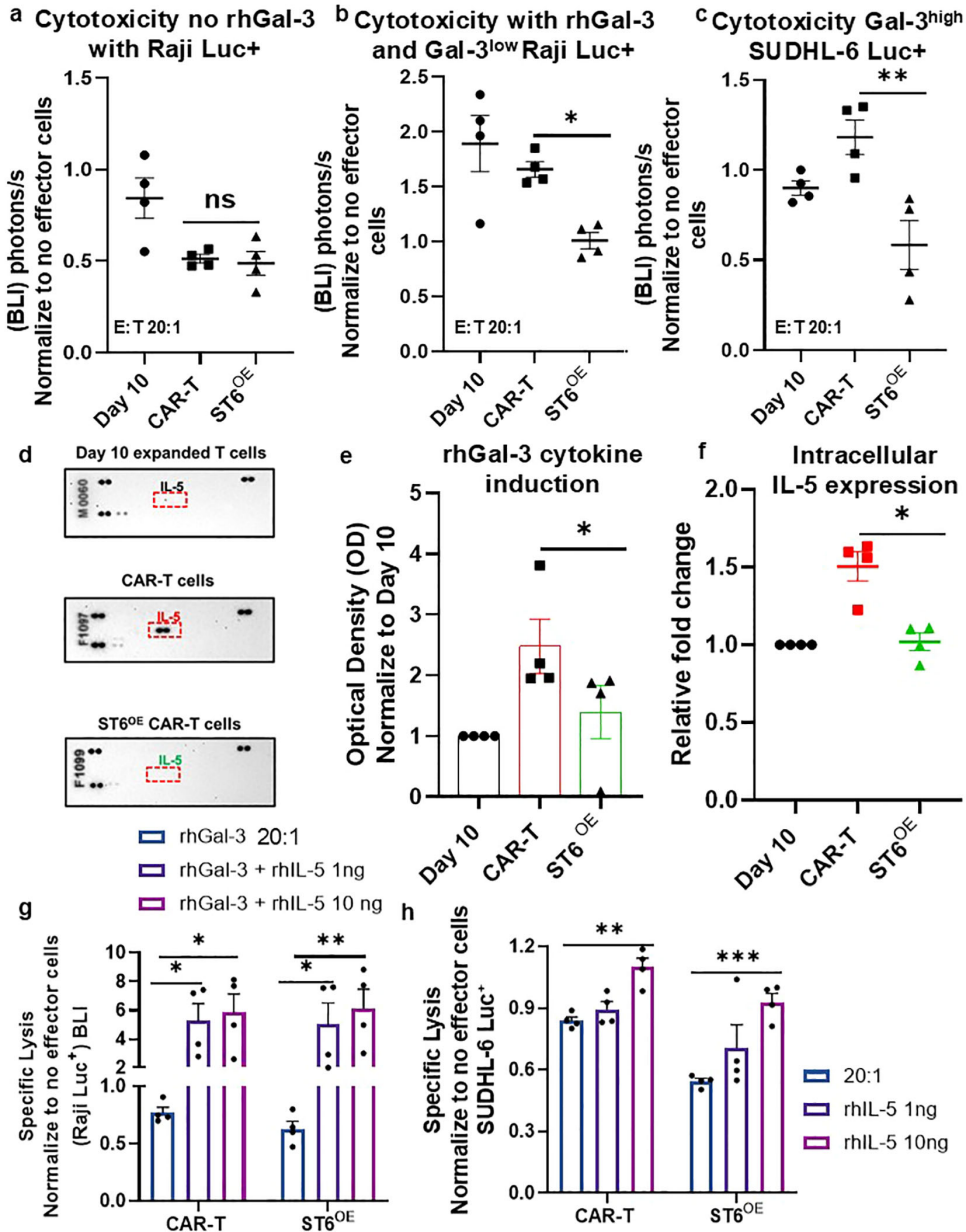
ST6^OE^ CAR-T cells exhibited effective tumoricidal activity in the presence of Gal-3 and evaded Gal-3-induced expression of IL-5, which compromised anti-tumor efficacy. Day 10-expanded T cells, CAR-T cells, and ST6^OE^ CAR-T cells were incubated with Gal-3^low^ Raji-luc^+^ cells or Gal-3^high^ SUDHL-6-luc^+^ cells at effector:target (E:T) ratios of 20:1. Raji cell cytotoxicity was evaluated **(a)** without rhGal-3 or **(b)** with rhGal-3, and **(c)** Gal-3^high^ SUDHL-6 cell cytotoxicity was examined without exogenous rhGal-3. Data are presented from n = 4 independent donors and analyzed using a two-way repeated-measures ANOVA with Sidak’s multiple-comparisons test, with **p < 0.01, *p < 0.05. **(d)** Human cytokine proteome profiling of supernatants from Day 10-expanded T cells, CAR-T cells, and ST6^OE^ CAR-T cells incubated with rhGal-3 for 16h revealed a marked upregulation of IL-5 (red hatched box) in CAR-T cells that was absent in ST6^OE^ CAR-T cells. **(e)** Arrays were repeated 4 times and graphed as Relative Optical Density (Mean ± SEM). Intracellular IL-5 expression was assessed by flow cytometry in control Day 10-expanded T cells, CAR-T cells and ST6^OE^ CAR-T cells after rhGal-3 incubation for 16h and mean ± SEM fold change from n = 4 independent donors was graphed **(f)** (*p < 0.05). Statistical significance was determined using a Mann–Whitney U test. CAR-T cells and ST6^OE^ CAR-T cells were incubated with **(g)** Gal-3^low^ Raji-luc^+^ cells or **(h)** Gal-3^high^ SUDHL6-luc^+^ at E:T ratios of 20:1 (**p <0.01, ***p <0.001). Statistical significance was determined using two-way repeated-measures ANOVA with Sidak’s multiple-comparisons test.

To assess the tumor-killing capacity of ST6^OE^ CAR-T cells vs. control anti-CD19 CAR-T cells, we performed a luciferase-based cytotoxicity assay using CD19^+^ Gal-3^low^ Raji-luciferase (luc)^+^ and Gal-3^high^ SUDHL-6-luc^+^ lymphoma cells (**Supplementary Figure 3**). CAR-T cells and lymphoma cells were co-cultured at effector: target (E:T) ratio of 20:1 for 16h with or without 4µg/mL rhGal-3. Post-incubation, CAR-T cells and Raji-luc^+^ co-cultures were lysed using Bright-GLO reagent and luc^+^ tumor viability was measured by bioluminescence imaging (BLI) detection, whose signal was directly proportional to the number of viable luc^+^ target tumor cells and therefore served as an inverse indicator of CAR-T cell-mediated cytotoxicity. In Gal-3-free conditions, both ST6^OE^ CAR-T cells and control CAR-T cells demonstrated comparable levels of Gal-3^low^ Raji-luc^+^ tumor cell killing (**Figure 5a**), indicating that enforced *ST6GAL1* did not compromise intrinsic cytotoxic function and baseline tumoricidal activity. However, upon Gal-3 exposure to both Gal-3^low^ Raji-luc^+^ cells and Gal-3^high^ SUDHL-6-luc^+^ lymphoma cells, a divergence in cytolytic activity was observed. Compared with control CAR-T cells, ST6^OE^ CAR-T cells exhibited an improved tumoricidal activity, as evidenced by lower BLI signal (*p* < 0.05 and *p* < 0.01, respectively), indicating functional resistance to Gal-3-mediated immunosuppression (**Figures 5b, c**). These findings confirmed that enforced *ST6GAL1* expression can help protect CAR-T cells from Gal-3-induced immunoregulation, even under conditions of high Gal-3 expression.

Next, we investigated the effect of Gal-3 stimulation on anti-CD19 CAR-T cell cytokine responses. Upon Gal-3 exposure, CAR-T cells exhibited a notable increase in IL-5 secretion, identifying a Gal-3–associated cytokine response in CAR-T cells (**Figure 5d**). This IL-5 induction was significantly attenuated in ST6^OE^ CAR-T cells, as confirmed by cytokine profiling (*p* < 0.05) (**Figure 5e**) and further validated by intracellular flow cytometry, indicating that enforced *ST6GAL1* expression limits Gal-3–driven cytokine skewing (**Figure 5f**) (*p* < 0.05). These findings suggest that IL-5 production occurs downstream of Gal-3 engagement and reflects a secondary immunomodulatory consequence of Gal-3 binding rather than a primary initiating mechanism.

Although IL-5 has been associated with eosinophil recruitment and immune modulation in clinical settings, its functional impact on CAR-T cells appears context dependent (31–33). To determine whether IL-5 directly contributed to Gal-3-mediated CAR-T cell dysfunction, cytotoxicity assays were performed in the presence of exogenous rhIL-5. Treatment of Raji-luc^+^ cell/SUDHL-6-luc^+^: T cell co-cultures with rhIL-5 and/or rhGal-3 showed that *ST6GAL1* overexpression does not confer resistance to IL-5–mediated suppression, as evidenced by increased BLI (*p* < 0.01 and *p* < 0.05, respectively) (**Figures 5g, h**). Together, these data demonstrated that *ST6GAL1*-mediated glycoengineering protects CAR-T cells by limiting upstream Gal-3 engagement and its downstream apoptotic and cytokine-skewing effects but does not preserve CAR-T cell function from IL-5 effects.

### 3.6 ST6^OE^ CAR-T cell treatment exhibited enhanced tumor control, mouse survival, and persistence *in vivo*

**Figure 6:**
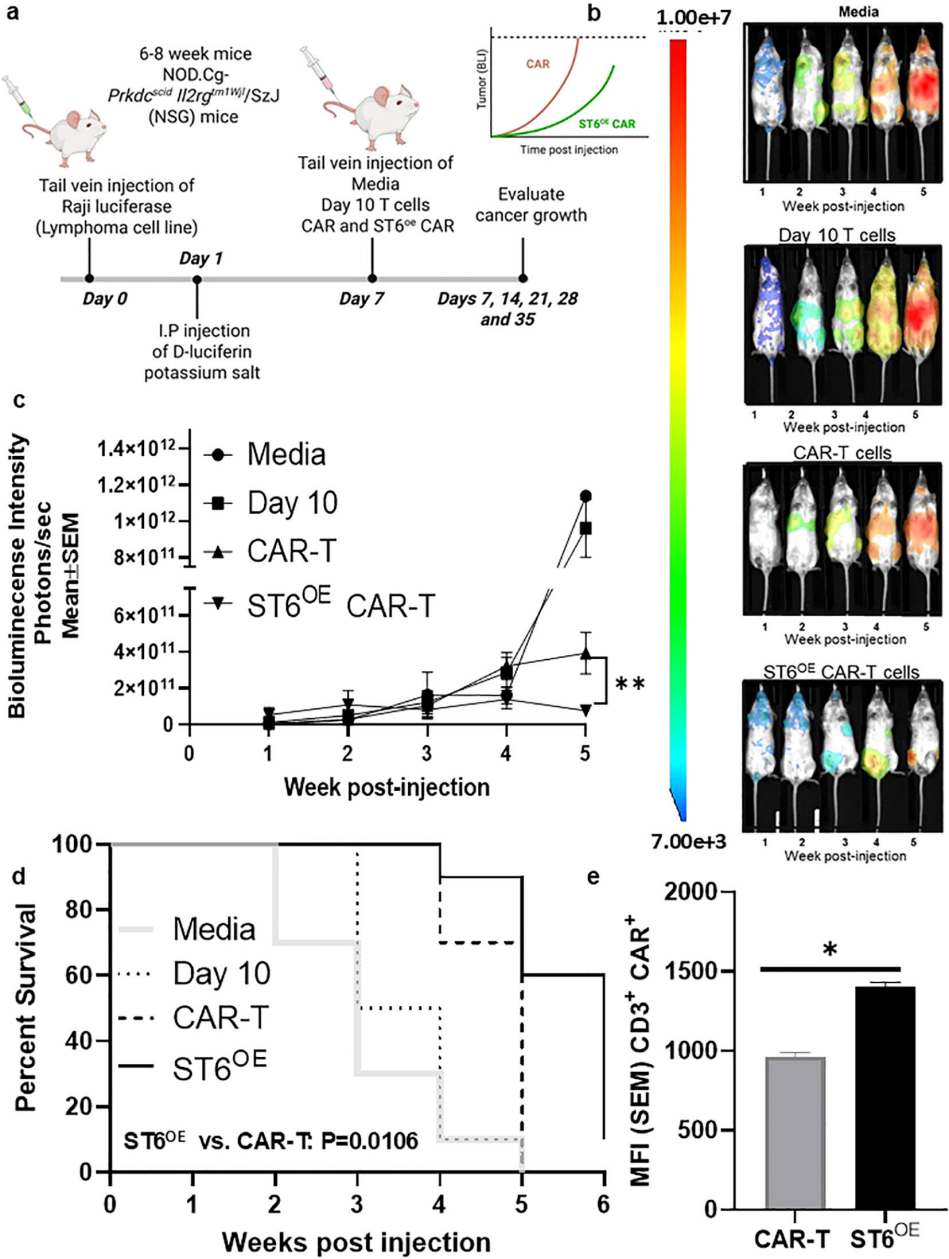
ST6^OE^ CAR-T cells exhibited potent *in vivo* anti-tumor activity and persisted longer *in vivo* than conventional CAR-T cells. Using a DLBCL xenograft model **(a)**, NSG mice were inoculated with Raji-luc^+^ cells and, after 7 days, treated with media (control), Day 10-expanded T cells, CAR-T cells, or ST6^OE^ CAR-T cells. Tumor growth in NSG mice was monitored weekly by *in vivo* BL imaging system **(b)**, and BLI measurements reflecting tumor burden from respective groups were graphed as shown (n = 9 mice per group) (**p < 0.01) **(c)**. Xenografted mouse survival data were graphed as shown (Gehan-Breslow-Wilcoxon test – **p = 0.0106) **(d)**, and flow cytometry of CAR^+^ T cells isolated from xenografted mouse spleens expressed as Mean ± SEM were graphed as shown (Mann–Whitney U test- *p<0.05) **(e)**.

To evaluate *in vivo* efficacy of ST6^OE^ CAR-T cells, we employed a NOD-SCID IL-2Rγ^-/-^ (NSG) mouse model grafted with CD19^+^ Raji-luc^+^ lymphoma. As depicted in **Figure 6a**, mice were injected i.v. with tumor cells on Day 0 and treated i.v. on Day 7 with either media, day 10 T cells, anti-CD19 ST6^OE^ CAR-T cells or control anti-CD19 CAR-T cells. Tumor progression was monitored by BLI detection weekly using AMI HT Imager System until 35 days post-treatment (**Figure 6b**). BLI results revealed significantly reduced tumor growth in mice treated with ST6^OE^ CAR-T cells compared to those treated with control CAR-T cells (*p* < 0.01) (**Figure 6c**). Kaplan-Meier survival curves showed improved overall survival in the ST6^OE^ CD19 CAR-T cell treatment group compared to control CAR-T cells (*p* = 0.011) (**Figure 6d**). CAR-T cell persistence was also assessed by flow cytometric analysis of splenocytes collected post-mortem. Spleens were harvested, mechanically dissociated, and stained with CAR-specific Abs to identify CAR-T cells. ST6^OE^ CAR T cells were detected at a significantly higher frequency in the spleen compared with control CAR-T cells (*p* < 0.05) (**Figure 6e**), suggesting that enforced *ST6GAL1* expression enhanced *in vivo* persistence of CAR-T cells. In all, these results indicated that ST6^OE^ CAR-T cells exhibited improved antitumor activity and persistence in this xenograft model. Importantly, this *in vivo* model was used to assess CAR-T cell persistence and antitumor efficacy following ST6GAL1 glycoengineering, rather than to directly test Gal-3 dependence *in vivo*.

## 4 Discussion

Despite clinical successes of CD19-targeted CAR-T cell therapies in treating r/r B-cell malignancies, durable remissions remain elusive for a substantial fraction of patients. A major potential obstacle is the tumor-promoting, immunosuppressive TME, wherein Gal-1, -3, and/or -9 can hinder T cell survival and function (12). In this investigation, we identified Gal-3 as a dominant extrinsic suppressor of anti-CD19 CAR-T cell activity and our studies demonstrated that CAR-T cells possessed intrinsic glycan features that render them particularly susceptible to Gal-3-mediated immunoregulation. Consistent with prior reports on galectin-mediated impairment of T cell survival and persistence, our data showed that CAR-T cells exhibited robust Gal-3-binding, leading to increased apoptosis and impaired tumor cell killing, highlighting the importance of strategies to block Gal-3-mediated suppression.

Our glycomics and glycosyltransferase assessments on CAR-T cells illuminated a signature glycome featuring Gal-3 ligand^high^ (α2,6 sialylation^low^/poly-LacNAc^high^) N-glycan expression likely regulated by elevations in *MGAT5*/*B4GALT1*/*GCNT2* N-glycan-modifying enzymes and concomitant suppressed levels of Gal-3 ligand-blocking enzyme, *ST6GAL1*. Our data showed that enforcing *ST6GAL1*/α2,6 sialylation on CAR-T cells blocked Gal-3 ligand activity, and significantly reduced Gal-3-mediated apoptosis and immunomodulation, resulting in improved CAR-T cell persistence and antitumor efficacy. These findings align with previous studies showing that α2,6 sialylation sterically impedes Gal-1 (34) and Gal-3 ligand activity (29, 35) and related cellular dysfunction (36, 37). In support of the broader relevance of N-glycan remodeling, recent work shows enhanced CAR-T efficacy following *MGAT5* silencing, further underscoring the therapeutic potential of glycoengineering strategies (38).

Given that T cell activation is known to induce substantial glycan remodeling, we observed that CAR-T cell glycomes were largely similar to those of non-transduced, Day-10-expanded T cells. This suggested that CAR transduction itself does not radically alter the global glycome, but instead fine-tunes glycosyltransferase expression in ways that have considerable functional consequences. These differences likely reflected the profound metabolic and transcriptional reprogramming that occurs during CAR-T manufacturing, where sustained CD3/CD28 activation and IL-2-driven proliferation promote aerobic glycolysis, enhance flux through the hexosamine biosynthetic pathway, and alter intracellular pools of UDP-GlcNAc and CMP–sialic acid pools that support N- and O-glycan synthesis (39, 40). Such metabolic remodeling is known to regulate Golgi-resident enzymes including *ST6GAL1*, *B4GALT1*, and *MGAT5*, thereby shaping the T cell glycome (41–43). These activation-associated glycome changes provide a plausible explanation for the reduced *ST6GAL1*/α2,6 sialylation expression and heightened galectin-binding capacity observed in CAR-T cells. Whether similar glycan vulnerabilities arise in CAR-T cell products incorporating alternative antigen targets or costimulatory domains remains an important area for future investigation.

In addition to promoting apoptosis, we observed that Gal-3 exposure skewed CAR-T cell cytokine production toward increased IL-5 secretion and that IL-5 directly compromised CAR-T cell cytotoxic function *in vitro*. While IL-5 is classically associated with eosinophil recruitment and type 2 immune responses, its role in cancer immunity is context-dependent (31, 44). Although IL-5-associated eosinophilia has been linked to favorable CAR-T cell outcomes in leukemia patients (45), our data suggested that Gal-3-induced IL-5 production may reflect a dysfunctional or maladaptive transcriptional state that directly impairs CAR-T cell effector function. The functional consequences of Gal-3 exposure likely depend on spatial and temporal Gal-3 concentrations within the TME, where localized high-density Gal-3 deposits may induce apoptosis, whereas lower concentrations may subtly reprogram cytokine output (46, 47). These observations highlight the complexity of galectin-mediated regulation and underscore the need to consider both dose and context when evaluating Gal-3-driven immune effects.

There are various approaches currently being explored to augment CAR-T cell efficacy, including direct optimization of CAR constructs and innovative combination strategies (48, 49). Glycoengineering has recently emerged as a complementary strategy, exemplified by enforced α1,3 fucosylation to enhance E-selectin ligand expression and tumor infiltration of CAR-T cells (50, 51). Our findings extend this paradigm by identifying Gal-3-mediated immunosuppression as a critical barrier to CAR-T cell persistence in DLBCL. Notably, Gal-3 is unlikely to be the sole lectin influencing CAR-T cell function: enhanced α2,6 sialylation may also modulate interactions with other glycan-binding receptors, such as Gal-1 or Siglecs, which are established regulators of immune signaling (13). Supporting the broader applicability of this approach, enforced *ST6GAL1* expression has been shown to enhance NK cell-mediated killing of CD22^high^ B-cell malignancies through improved Siglec-2 (CD22) interactions (52–54), suggesting that *ST6GAL1*-mediated glycoengineering may confer multifaceted benefits across immune effector cell types.

**Figure 7:**
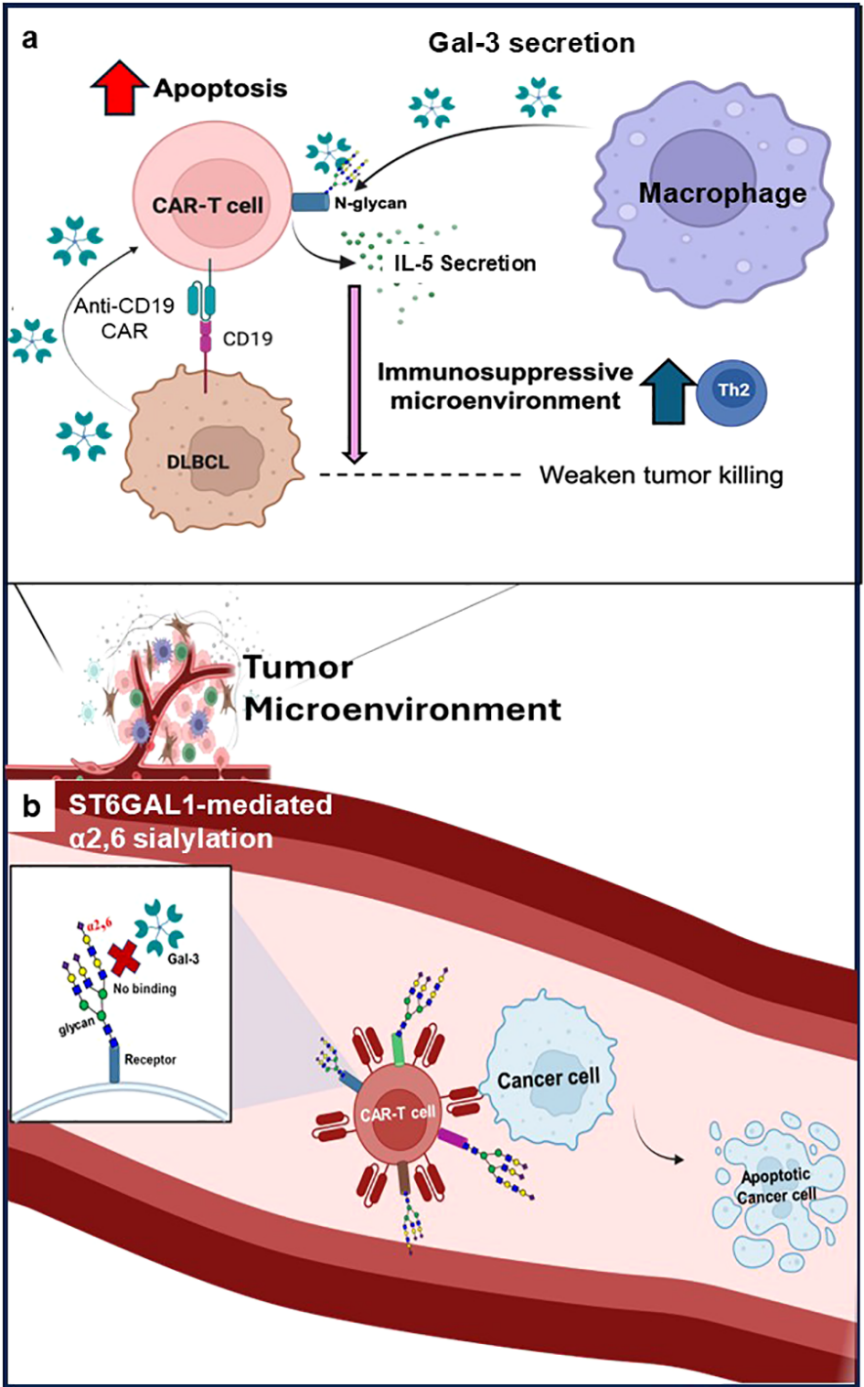
**(a)** A cartoon model depicting the impact of Gal-3 on anti-tumor CAR-T cell function and **(b)** the protective role of enforced *ST6GAL1*/α2,6 sialylation on Gal-3-dependent CAR-T cell immunoregulation.

In summary, our data demonstrate that CAR-T cells are intrinsically vulnerable to Gal-3-mediated immunoregulation due to activation-induced- and manufacturing-associated remodeling of their glycome and glycan biosynthetic machinery. We propose that enforcing α2,6 sialylation through *ST6GAL1* shields CAR-T cells from Gal-3-induced apoptosis and maladaptive cytokine skewing, resulting in enhanced persistence and antitumor efficacy (**Figures 7a, b**). This glycoengineering strategy offers a promising new avenue and perhaps provides proof-of-concept to devise more targeted small molecule inhibitors to individual galectins to improve CAR-T cell durability in DLBCL and potentially other malignancies characterized by Gal-1 and/or Gal-3 immunosuppressive microenvironments. While Gal-3 is reported to be expressed by lymphoma cells and components of the tumor microenvironment, Gal-3 levels were not directly quantified or manipulated in the *in vivo* xenograft model used here. Accordingly, the enhanced persistence and anti-tumor activity observed with *ST6GAL1*-overexpressing CAR-T cells should be interpreted as evidence of improved CAR-T cell fitness *in vivo*, rather than direct evidence of Gal-3-dependent suppression in this setting. Mechanistic attribution to Gal-3 is supported primarily by extensive *in vitro* binding, apoptosis, and functional assays.

## Data Availability

The original contributions presented in the study are included in the article/**Supplementary Material**, further inquiries can be directed to the corresponding author/s.
