# Supplementary material for: Glycoengineering CAR-T cells to overcome galectin-3-mediated immunosuppression
Source: Front Immunol. 2026 Feb 18;17:1766555. doi: 10.3389/fimmu.2026.1766555 (PMC12956802; doi:10.3389/fimmu.2026.1766555)
Supplement: Supplementary file 1 [file Table1.docx]

**SUPPLEMENTARY TABLES**

**Supplementary Table 1**. Antibodies and reagents used for ELISA, RT-qPCR, flow cytometry, isolation/transduction/expansion of human T cells, lentiviral production and *in vivo* experiments.

| **Antibodies/Reagents** | **Source** | **Identifier** |
| --- | --- | --- |
| Human Galectin-1 Quantikine ELISA Kit | R&D Systems™ | Cat# DGAL10 |
| Human Galectin-3 Quantikine ELISA Kit | R&D Systems™ | Cat# DGAL30 |
| Human Galectin-9 Quantikine ELISA Kit | R&D Systems™ | Cat# DGAL90 |
| Human ST6GAL1 TaqMan® primer | Thermo Scientific | AssayID: Hs00949382_m1 |
| Human ST3GAL1 TaqMan® primer | Thermo Scientific | AssayID: Hs00161688 |
| Human B4GALT1 TaqMan® primer | Thermo Scientific | AssayID: Hs00155245_m1 |
| Human MGAT4 TaqMan® primer | Thermo Scientific | AssayID: Hs00608465 |
| Human MGAT5 TaqMan® primer | Thermo Scientific | AssayID: Hs00159136_m1 |
| Human GCNT1 TaqMan® primer | Thermo Scientific | AssayID: Hs01922706_s1 |
| Human GCNT2 TaqMan® primer | Thermo Scientific | AssayID: Hs00377334_m1 |
| Human 18s TaqMan® primer | Thermo Scientific | AssayID: Hs03003631_g1 |
| Applied Biosystems™ TaqMan™ Fast Advanced Master Mix | Thermo Scientific | Cat# 4444556 |
| TaqMan® Fast Advanced Master Mix | Thermo Scientific | Cat# 4444557 |
| RNeasy Plus Mini Kit (250) | QIAGEN | Cat# 74136 |
| SuperScript™ VILO™ cDNA Synthesis Kit | Invitrogen™ | Cat# 11-754-050 |
| Biotin-SP (long spacer) AffinityPure Goat Anti-Mouse IgG, F(ab')₂ fragment specific | Jackson ImmunoResearch Laboratories | Cat # 115-065-072 |
| Alexa Fluor® 647 Rat IgG2a, κ Isotype Ctrl Antibody | Biolegend | Cat# 400526 |
| LIVE/DEAD™ Fixable Aqua Dead Cell Stain Kit, for 600 nm excitation | Fisher | Cat# 50-112-1525 |
| PE anti-human CD3 Antibody | Biolegend | Cat# 300408 |
| FITC anti-human CD4 Antibody | Biolegend | Cat# 317408 |
| PerCP anti-human CD8a Antibody | Biolegend | Cat# 300922 |
| Dead Cell Apoptosis Kit with Annexin V Alexa Fluor™ 488 & Propidium Iodide (PI) | Fisher | Cat# V13241 |
| PE Streptavidin (High Concentration) | Fisher | Cat # 405245 |
| Zombie Aqua™ Fixable Viability Kit | Biolegend | Cat # 423101 |
| Alexa Fluor® 647 anti-mouse/human Mac-2 (Galectin-3) Antibody | Biolegend | Cat# 125408 |
| APC anti-human Galectin-9 Antibody | Biolegend | Cat# 348908 |
| Anti-human Galectin-1 antibody | Abcam | Cat# ab25138 |
| APC Mouse IgG1, κ Isotype Ctrl Antibody | Biolegend | Cat# 400120 |
| Biotin Rat IgG2a, κ Isotype Ctrl Antibody | Biolegend | Cat# 400504 |
| Brilliant Violet 421™ Rat IgG2b, κ Isotype Ctrl Antibody | Biolegend | Cat# 400640 |
| FITC Mouse IgG1, k Isotype Ctrl (FC) | Biolegend | Cat# 981802 |
| PerCP Mouse IgG1, k Isotype Ctrl | Biolegend | Cat# 400148 |
| PE Mouse IgG2a, k Isotype Ctrl (FC) | Biolegend | Cat# 400212 |
| APC anti-mouse/human IL-5 | Biolegend | Cat# 504306 |
| Paraformaldehyde Solution | Thermo Scientific | Cat# NC1660551 |
| Solanum Tuberosum (Potato) Lectin (STL, PL), Unconjugated | Vector Laboratories | Cat# L-1160-5 |
| *Maackia Amurensis* Lectin II (MAL II), Biotinylated | Vector Laboratories | Cat# B-1265 |
| *Sambucus Nigra* Lectin (SNA), Fluorescein | Vector Laboratories | Cat# FL-13012 |
| Recombinant human galectin-1 (rhGal1) | R&D Systems™ | Cat# 1152-GA-050 |
| Recombinant human galectin-3 (rhGal3) | R&D Systems™ | Cat# 1154-GA-050 |
| Recombinant human galectin-9 (rhGal9) | R&D Systems™ | Cat# 2045GA050 |
| Recombinant Human IL-5 Protein | R&D Systems™ | Cat# 205-IL-005/CF |
| Human IL-2 Recombinant Protein | R&D Systems™ | Cat# 202-IL-050/CF |
| ImmunoCult™ Human CD3/CD28 T Cell Activator | Stem Cell Technologies | Cat# 10971 |
| Naive CD4+ T Cell Isolation Kit II, human | Miltenyi Biotec | Cat# 130-094-131 |
| Naive CD8+ T Cell Isolation Kit, human | Miltenyi Biotec | Cat# 130-093-244 |
| Pan T Cell Isolation Kit, human | Miltenyi Biotec | Cat # 130-096-535 |
| HiPure Plasmid Filter Maxiprep Kit | Invitrogen™ | Cat# K210017 |
| ViraPower™ Lentiviral Gateway™ Expression Kit | Invitrogen™ | Cat# K496000 |
| One Shot Stbl3 E.coli | Invitrogen™ | Cat# C7373-03 |
| Lipofectamine 2000 | Invitrogen™ | Cat# 11668027 |
| Bright-Glo™ Luciferase Assay System | Promega | Cat# E2610 |
| VivoGlo™ Luciferin, In Vivo Grade | Promega | Cat# P1042 |
| Firefly Luciferase Lentivirus (Puromycin) | BPS Bioscience | Cat# 79902-P |
| Expression Negative Control Lentivirus (Puromycin) | BPS Bioscience | Cat# 79692-P |
| Nano-Glo® Luciferase Assay System | Promega | Cat# N1110 |
| Nano-Glo® Fluorofurimazine In Vivo Substrate | Promega | Cat# [N4110](https://www.promega.com/catalogredirection.aspx?partno=n4110) |

**Supplementary Table 2.** Demographics and clinical features of patients with DLBCL and healthy controls

|  | Patients with DLBCL (n = 31) | Healthy Controls (n = 31) |
| --- | --- | --- |
| Sex, *n (%)*  Male  Female  Race, *n (%)*  African American  White  White Hispanic | 13 (41.9)  18 (58.1)  3 (9.7)  3 (9.7)  25 (80.6) | 12 (38.7)  19 (61.3)  3 (9.7)  12 (38.7)  16 (51.6) |
| Age at diagnosis, *y (mean ± SD)* | 63.5 ± 16.5 | 54.3 ± 13.2 |
| DLBCL stage, *n (%)*  Primary DLBCL  Stage I  Stage II  Metastatic DLBCL  Stage III  Stage IV  Treatment at Biopsy, *n (%)*  CAR-T Cell therapy  Chemotherapy  Chemotherapy and  Radiation | **10 (32.3)**  1 (3.2)  9 (29.1)  **21 (67.7)**  3 (9.6)  18 (58.1)  1 (3.2)  26 (83.9)  4 (12.9) |  |
