# Supplementary figures and images for: Glycoengineering CAR-T cells to overcome galectin-3-mediated immunosuppression
Source: Front Immunol. 2026 Feb 18;17:1766555. doi: 10.3389/fimmu.2026.1766555 (PMC12956802; doi:10.3389/fimmu.2026.1766555)

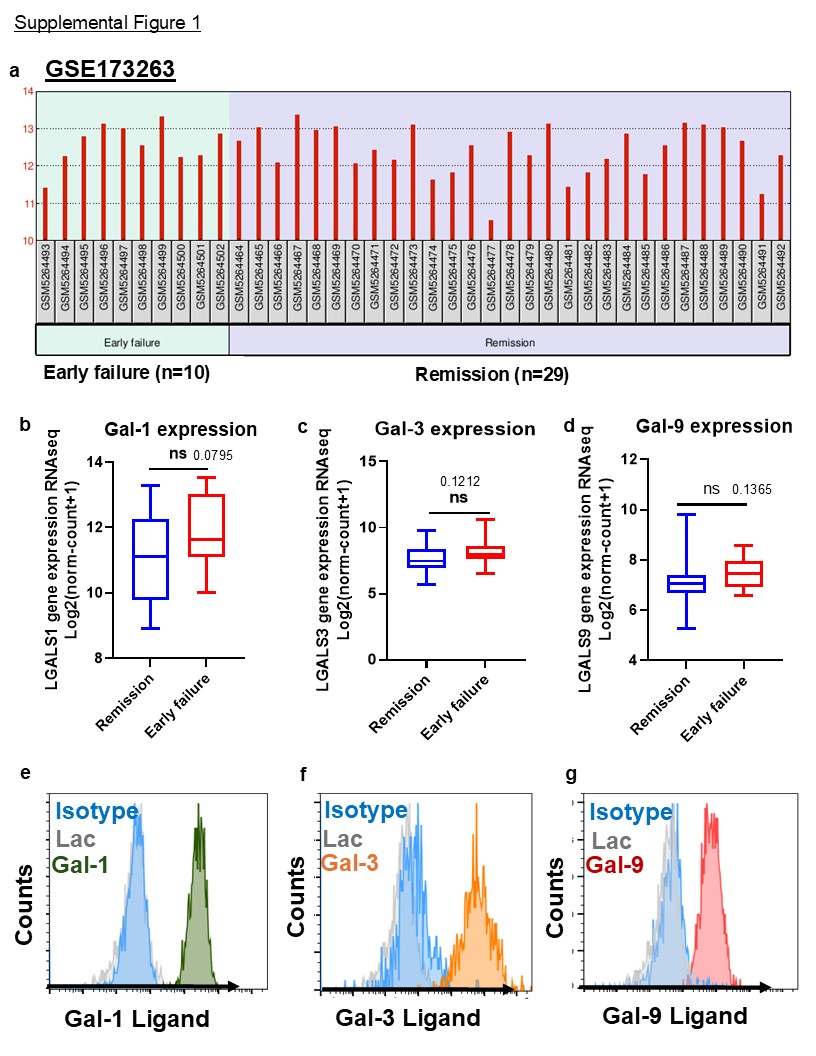

Supplement: Supplementary file 2 [file Image1.jpeg]

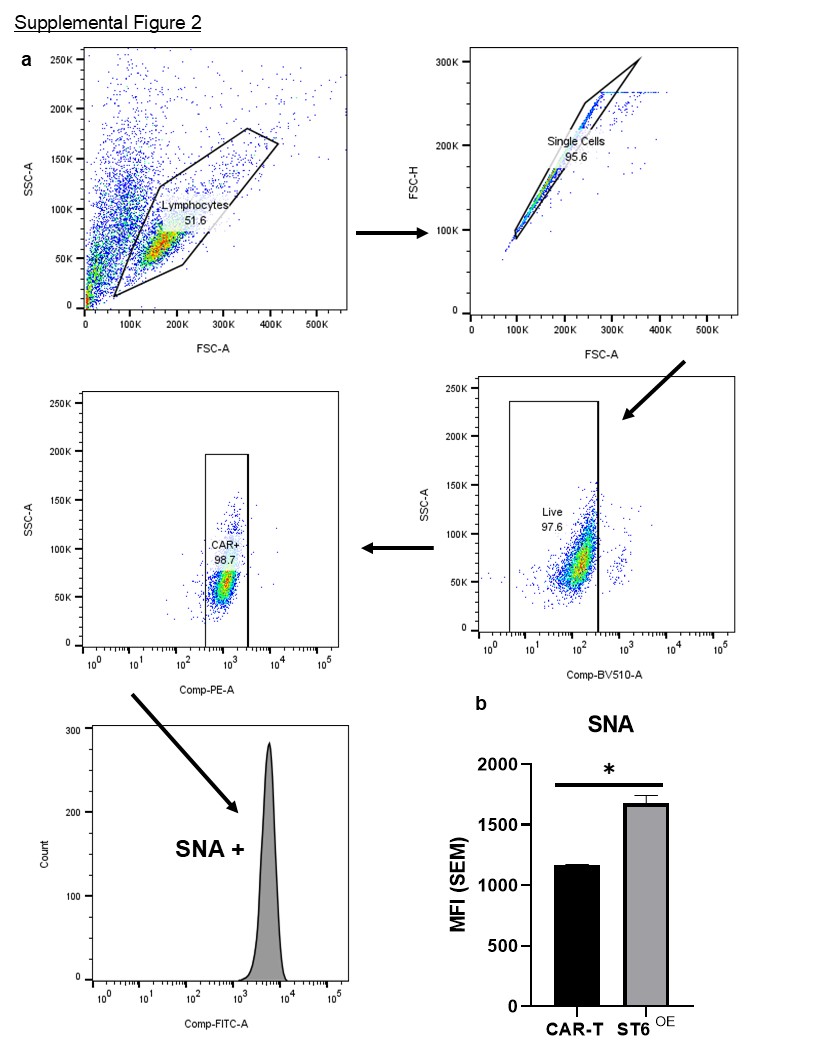

Supplement: Supplementary file 3 [file Image2.jpeg]

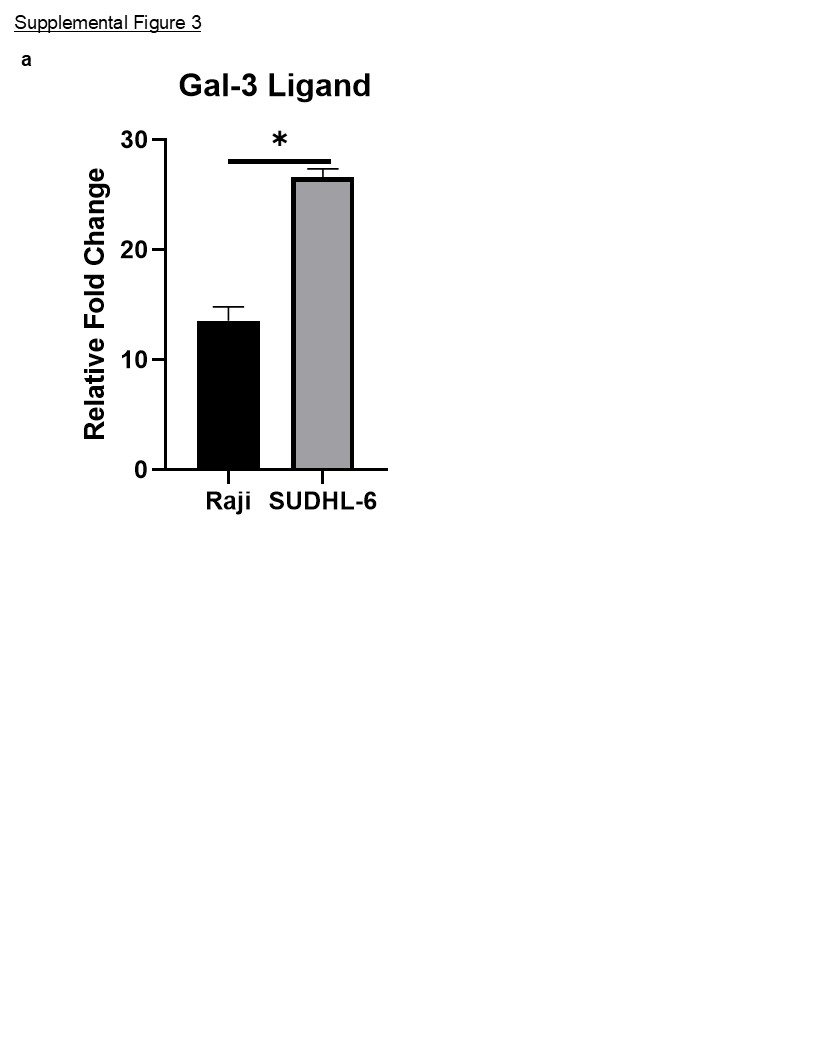

Supplement: Supplementary file 4 [file Image3.jpeg]

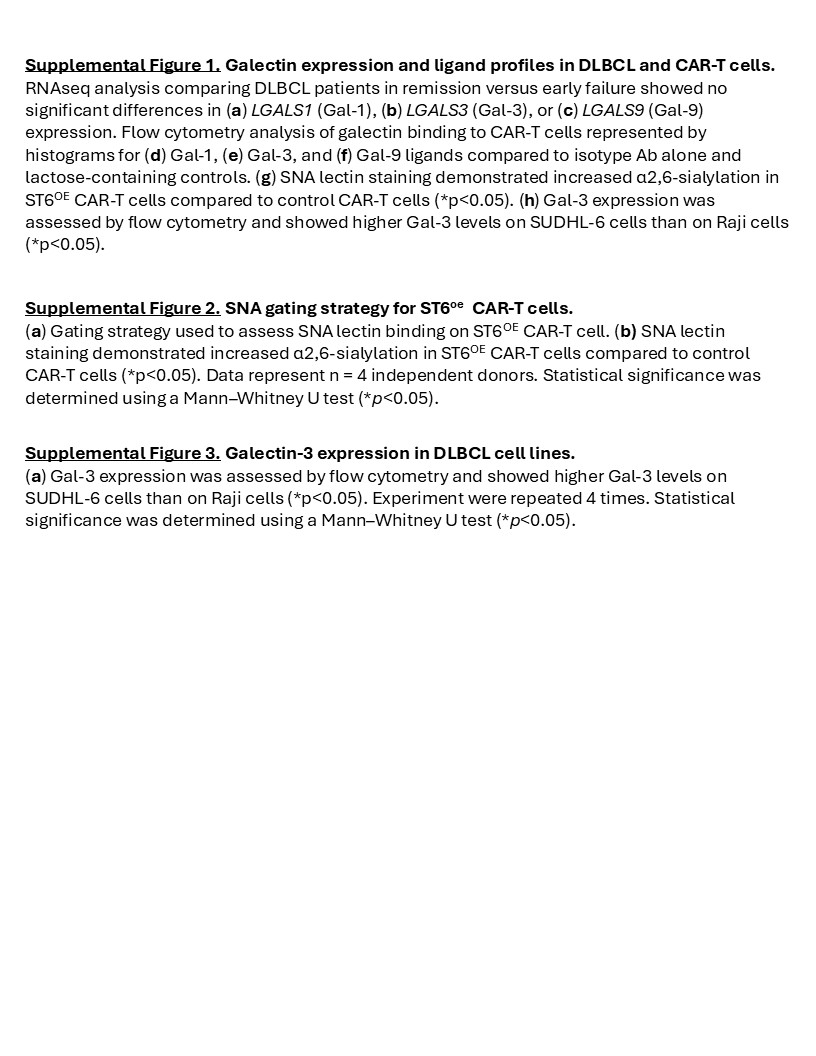

Supplement: Supplementary file 5 [file Image4.jpeg]
